# Berberine-releasing electrospun scaffold induces osteogenic differentiation of DPSCs and accelerates bone repair

**DOI:** 10.1038/s41598-020-79734-9

**Published:** 2021-01-13

**Authors:** Lan Ma, Yijun Yu, Hanxiao Liu, Weibin Sun, Zitong Lin, Chao Liu, Leiying Miao

**Affiliations:** 1 Department of Cariology and Endodontics, Nanjing Stomatological Hospital, Medical School of Nanjing University, Nanjing, 210093 China; 2 Department of Periodontology, Nanjing Stomatological Hospital, Medical School of Nanjing University, Nanjing, 210093 China; 3 Department of Dentomaxillofacial Radiology, Nanjing Stomatological Hospital, Medical School of Nanjing University, Nanjing, 210093 China; 4 Department of Orthodontics, Nanjing Stomatological Hospital, Medical School of Nanjing University, Nanjing, 210093 China

**Keywords:** Biomaterials, Drug delivery

## Abstract

The repair of skeletal defects in maxillofacial region remains an intractable problem, the rising technology of bone tissue engineering provides a new strategy to solve it. Scaffolds, a crucial element of tissue engineering, must have favorable biocompatibility as well as osteoinductivity. In this study, we prepared berberine/polycaprolactone/collagen (BBR/PCL/COL) scaffolds with different concentrations of berberine (BBR) (25, 50, 75 and 100 μg/mL) through electrospinning. The influence of dosage on scaffold morphology, cell behavior and in vivo bone defect repair were systematically studied. The results indicated that scaffolds could release BBR stably for up to 27 days. Experiments in vitro showed that BBR/PCL/COL scaffolds had appropriate biocompatibility in the concentration of 25–75 μg/mL, and 50 and 75 μg/mL scaffolds could significantly promote osteogenic differentiation of dental pulp stem cells. Scaffold with 50 μg/mL BBR was implanted into the critical bone defect of rats to evaluate the ability of bone repair in vivo. It was found that BBR/PCL/COL scaffold performed more favorable than polycaprolactone/collagen (PCL/COL) scaffold. Overall, our study is the first to evaluate the capability of in vivo bone repair of BBR/PCL/COL electrospun scaffold. The results indicate that BBR/PCL/COL scaffold has prospective potential for tissue engineering applications in bone regeneration therapy.

## Introduction

Many diseases can lead to skeletal defects in maxillofacial region, such as traumatic incidents, inflammation and tumour. Although bone has remarkable self-healing capacity, it is insufficient for large volume skeletal defects repair.

The currently effective methods for reconstructing large skeletal defects include bone transport, bone grafting, and biomaterial implantation^1^. The emerging tissue engineering technology provides a novel approach for bone regeneration. Bone tissue engineering is a method of implanting a composite of seed cells, growth factors, and scaffold materials, which are well-known as three crucial elements, into defect site. With the degradation of the scaffold material, the released growth factors promote cell proliferation and differentiation to repair the defects^2,3^. The scaffold is served as support for new bone growth and provides a place for cell activity^4^. Thus, the ideal bone scaffold should have the property of appropriate biocompatibility, high porosity, adequate mechanical strength, and fabulous osteoinductivity^5,6^. The classic approach to induce cells osteogenic differentiation is by adding growth factors, such as VEGF, BMP2 and TGF-β^7,8^. However, the preloaded growth factors of the scaffold are fragile and easy to be degraded, it is hard to sustain the effective concentration at the target site for a long time, which limits their application. Therefore, the research of substitutes for osteoinductive growth factor has been concentrated.

Berberine is an isoquinoline alkaloid, an active component in the traditional Chinese medicine rhizoma coptidis, which can be easily extracted from herbs and is mainly used in the treatment of digestive system diseases such as diarrhea and gastroenteritis^9,10^. Numbers of studies have proved that berberine has various of pharmacological effects, such as anti-diabetes^11^, anti-inflammatory^12^, antibacterial^13^, anti-cancer^14^, and lowering blood lipids^15^. With further research, researchers have found that berberine has a significant role on bone protection. Xu et al^16^. detected that berberine has therapeutic effect on glucocorticoid-induced osteoporosis by inhibiting bone absorption and promoting osteogenesis. Tao et al^17^. investigated the mechanism of bone promoting effect of berberine on mesenchymal stem cells (MSCs) in vitro, and found that berberine induced osteoblast differentiation through the Wnt/β-catenin signaling pathway. Liu et al^18^. detected that berberine may promoted the osteogenic differentiation of human periodontal ligament stem cells (PDLSCs) by activating the intracellular ERK-FOS signalling pathways. Therefore, based on the previous studies, we envision that BBR may perform as a growth factor substitute to induces bone formation in vivo.

Over the past few decades, nanofibers in various forms have received extensive of attention^19^. They can fabricate tissue scaffolds of different shapes, sizes, and structures to fill anatomical defects and provide the structural and biological support for cell growth, proliferation, and differentiation. Electrospinning is a convenient method to manufacture nanofibers. The polymer solution is processed into uniform fibers with diameters ranging from nanometers to microns under electrostatic forces^20^. The fibers are stacked into a network architecture, which can mimic the characteristics of the extracellular matrix (ECM)^21^. Electrospun nanofiber scaffolds have been widely used in the sustained release of reagents, including antibiotics, growth factors, and plasmid DNA^22–24^. Inspired by this, we take the advantages of electrospinning technology to prepare the blended scaffold as the drug carrier of this study.

In this study, to obtain an ideal scaffold with suitable biocompatibility and osteoinductivity properties, berberine was loaded into the PCL/COL scaffold via electrospinning process. The physicochemical properties of the electrospun scaffolds were characterized. Furthermore, the effect of BBR/PCL/COL scaffolds on the osteogenic differentiation of DPSCs in vitro and its ability to repair bone defects in vivo were evaluated.

## Results

### Characteristics of scaffolds

**Figure 1:**
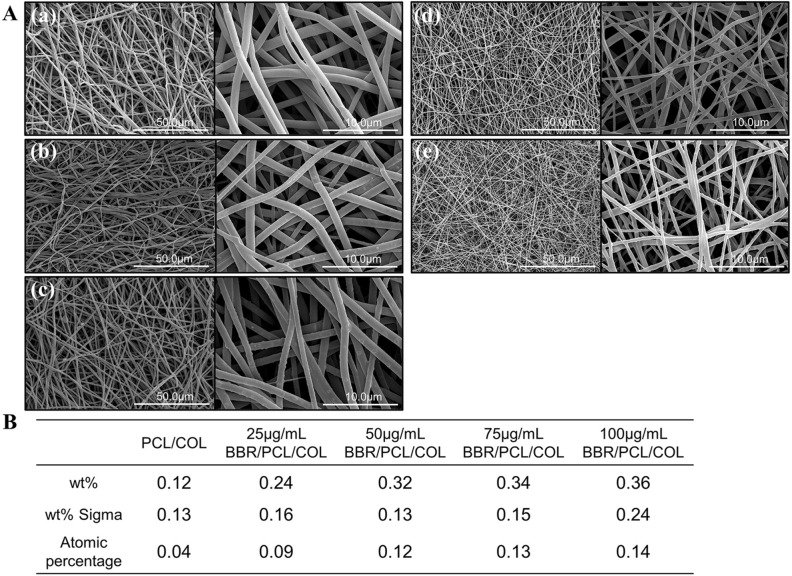
Scaffolds morphology and elemental composition. (**A**) SEM images of PCL/COL scaffold (**a**) and BBR/PCL/COL (25, 50, 75 and 100 μg/mL) scaffolds (**b**–**e**). (**B**) The Cl elemental compositions of scaffolds.

Scanning electron microscope (SEM) results in Fig. 1A shows that all scaffolds exhibited a high porous structure with fibers interconnected into a network. The PCL/COL scaffold fibers were uniform and smooth (Fig. 1A(a)). As for BBR/PCL/COL (25, 50, 75 and 100 μg/mL) scaffolds, uniform granular protrusions could be found on the surface of fiber, which were supposed to be the encapsulated BBR. The fiber diameter decreased with the BBR concentration increased (Fig. 1A(b, c, d, e)). The average diameter of fibers was 980.70 ± 159.32 nm, 1042.32 ± 156.40 nm, 945.32 ± 162.03 nm, 599.29 ± 175.59 nm, 468.43 ± 94.26 nm, respectively. The reason for the decrease in fiber diameter perhaps attributed to the change of solution conductivity and viscosity caused by BBR.

Energy dispersive x-ray spectroscopy (EDX) results in Fig. 1B shows all BBR/PCL/COL scaffolds contained Cl element, which was considered as the characteristic element of BBR. Cl element weight ratios were 0.24 wt%, 0.32 wt%, 0.34 wt% and 0.36 wt% for the four BBR/PCL/COL scaffolds. Although PCL/COL group detected the Cl element of 0.12 wt%, taking into account the error of 0.13, we believe that PCL/COL does not contain BBR.

**Figure 2:**
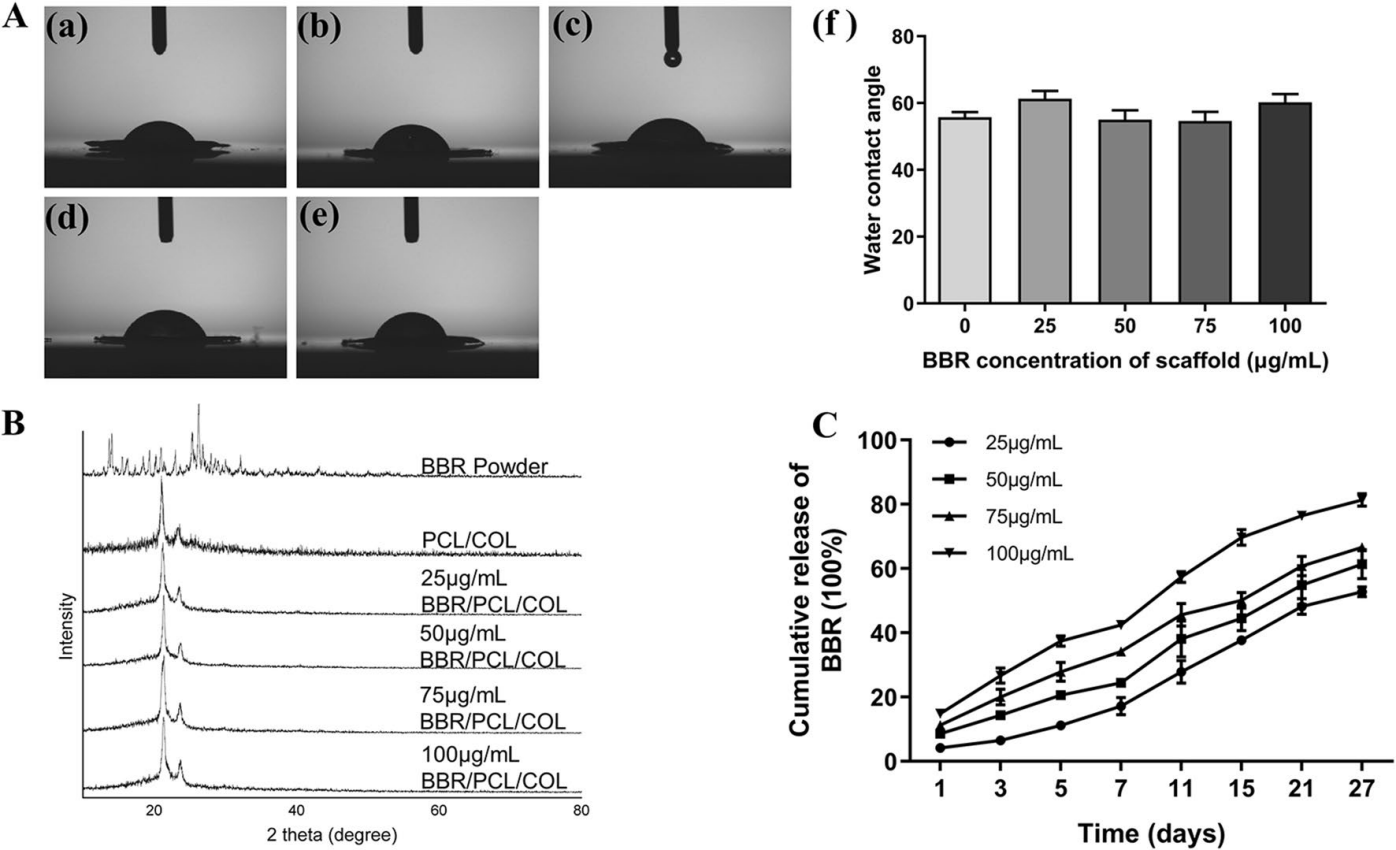
Characterization of scaffolds. (**A**) Water contact angle analysis of scaffolds. (**B**) XRD spectra of PCL/COL scaffold, BBR/PCL/COL (25, 50, 75 and 100 μg/mL) scaffolds and BBR powder. (**C**) Cumulative release results of BBR from the BBR/PCL/COL (25, 50, 75 and 100 μg/mL) scaffolds.

The results of the water contact angles were shown in Fig. 2A below, and the specific contact angle of PCL/COL scaffold was 55.76 ± 2.19°, while scaffolds with 25, 50, 75, 100 μg/mL BBR were 61.30 ± 3.32°, 55.00 ± 4.01°, 54.63 ± 3.87°, 60.23 ± 3.49°. No statistical difference was found between groups (*P* > 0.05). All the scaffolds had favorable hydrophilic properties which were conducive to cell adhesion and proliferation.

X-ray diffraction (XRD) was acquired to evaluate the composition of the scaffolds. As shown in Fig. 2B, compared to the PCL/COL scaffold, the position and intensity of the peaks in the XRD pattern of BBR/PCL/COL scaffolds did not change after loading the BBR. However, obvious difference was found between the XRD patterns of BBR powder and BBR/PCL/COL scaffolds. The results indicated that the combine of BBR did not change the original crystallinity of PCL and COL.

### Cumulative release of BBR from BBR/PCL/COL scaffolds

The cumulative release results of BBR/PCL /COL scaffold were shown in Fig. 2C. A low burst release was found on the first day in the high-concentration scaffold groups (50, 75, 100 μg/mL) (8.63% ± 0.50%, 11.31% ± 1.03% and 14.83% ± 1.69%, respectively). The initial release of the low-concentration scaffold groups (25 μg/mL) was 4.23% ± 0.27%, and all groups of scaffolds could release BBR stably for up to 27 days. On day 27, 52.8%, 61.4%, 66.6%, 81.4% of the total drug was released from BBR/PCL/COL scaffold, respectively. BBR/PCL/COL scaffold could be considered as a favorable drug carrier.

### The morphology of DPSCs cultured on scaffolds

**Figure 3:**
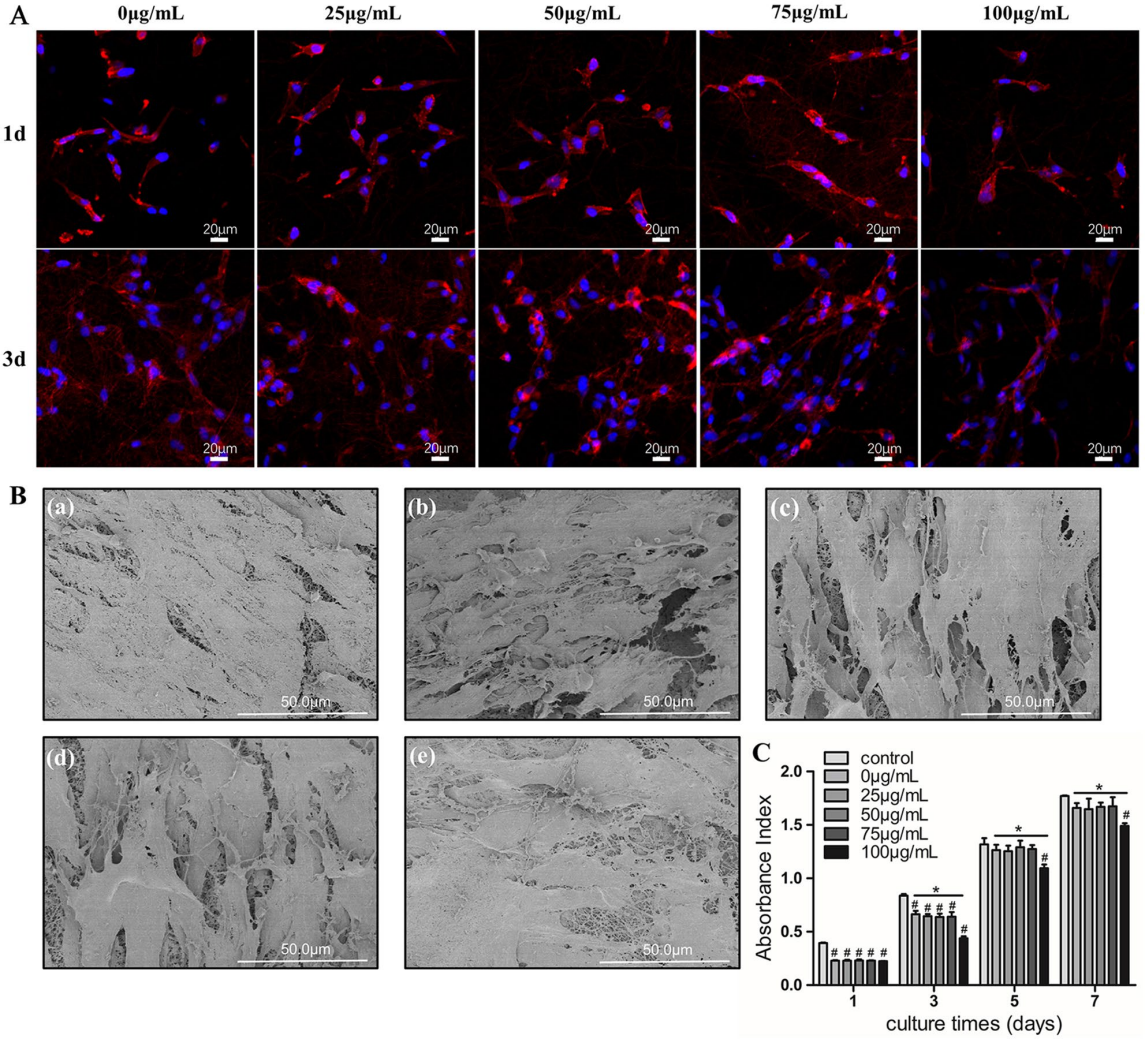
The morphology and proliferation of DPSCs cultured on scaffolds. (**A**) DAPI and actinred immunocytochemical staining of DPSCs co-cultured on scaffolds after 1 day and 3 days. (**B**) The morphology of DPSCs cultured on scaffolds for 7 days. (**a**) PCL/COL scaffold; (**b**, **c**, **d**, **e**) BBR/PCL/COL (25, 50, 75 and 100 μg/mL) scaffolds. (**C**) Proliferation rate of DPSCs cultured on the different scaffolds at days 1, 3, 5, 7. “#” means the comparison between the control group and the experimental group, *P* < 0.05; “∗” means the comparison between experimental groups, *P* < 0.05.

Immunocytochemical staining and SEM were used to observe the morphology of DPSCs cultured with scaffolds. We stained the samples with DAPI and actinred on day 1, 3. The DAPI blue-stained cell nucleus and the actinred red-stained cytoskeleton were observed under the confocal microscopy (Fig. 3A). On day 1, DPSCs of all groups adhered to the scaffolds and presented in spindle shape. On day 3, the number of nucleus significantly increased with the cytoplasm expanded to the surrounding, the cells further elongated and some cells gathered together. DPSCs showed uniform attachment in all scaffolds. After co-cultured for 7 days, the SEM images Fig. 3B shows that DPSCs were in admirable condition, cells extended the cilia into the fibers and fused together. The results confirmed that scaffolds had admirable biocompatibility.

### The proliferation of DPSCs cultured on scaffolds

The proliferation of DPSCs seeded on PCL/COL scaffold and BBR/PCL/COL (25, 50, 75 and 100 μg/mL) scaffolds were measured quantitatively by cell counting kit-8 (CCK8) assay. The cellular metabolism was detected on day 1, 3, 5, 7, and the result indicated that cellular proliferation was stable in all groups (Fig. 3C). On day 1 and 3 cellular proliferation in the scaffold groups were lower than control group, no significant difference was found between the experimental groups on day 1 and 3 (*P* > 0.05). The reason speculated was that the bottom of the plate was designed to facilitate cell adhesion, so the proliferation of cells occurred earlier. Compared with the PCL/COL scaffold group, the BBR/PCL/COL scaffold with the concentration of 100 μg/mL showed poor performance at day 3, 5 and 7 (*P* < 0.05), which may cause by the increasing concentration of drug. Therefore, we considered that DPSCs have favorable biocompatibility in PCL/COL scaffold and BBR/PCL/COL scaffolds with the BBR concentration of 25–75 μg/mL.

### Osteogenic differentiation of DPSCs cultured in scaffolds

**Figure 4:**
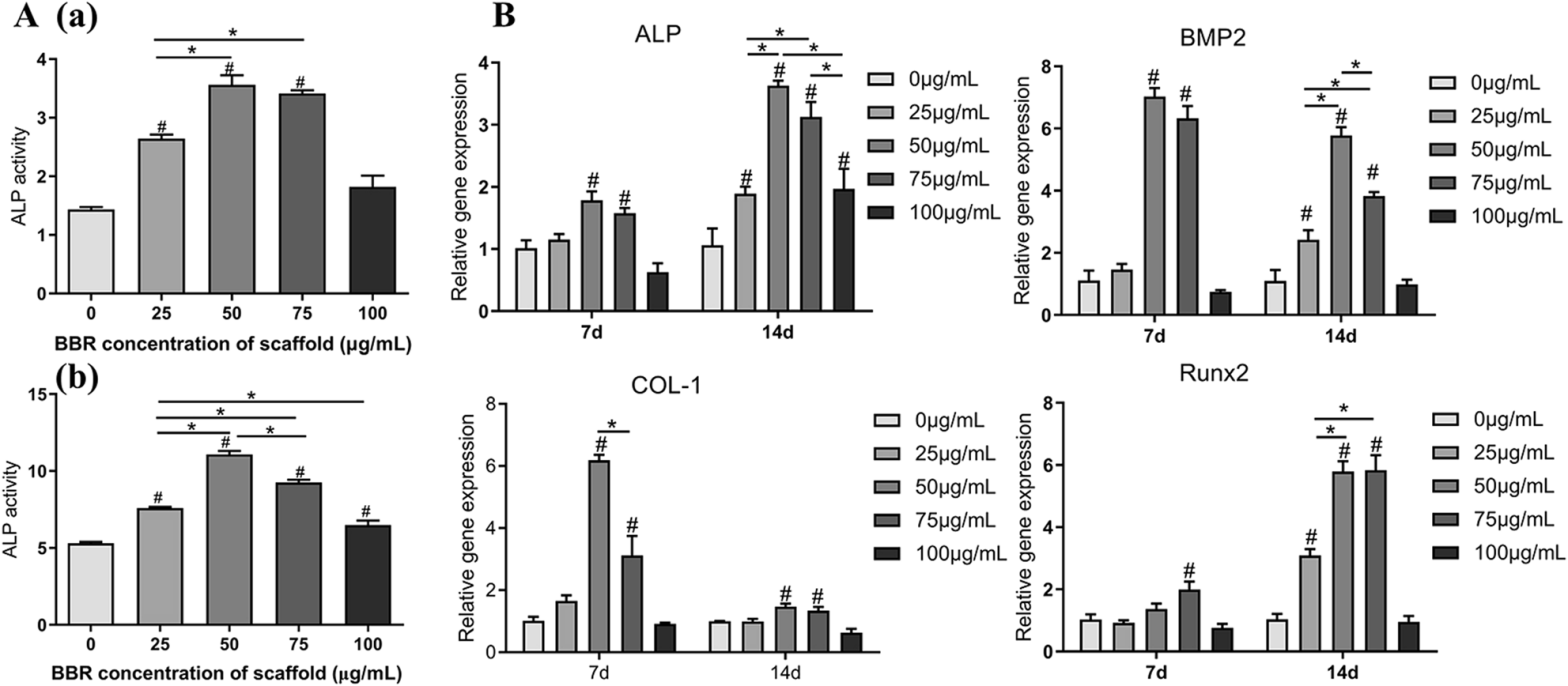
Osteogenic differentiation of DPSCs cultured on scaffolds. (**A**) ALP activity of DPSCs co-cultured on scaffolds after 7 days (**a**) and 14 days (**b**). (**B**) Gene expressions of ALP, BMP2, COL-1, Runx2 in DPSCs co-cultured on the scaffolds after 7 days and 14 days. “#” means the comparison between the control group and the experimental group, *P* < 0.05; “∗” means the comparison between experimental groups, *P* < 0.05.

In order to investigate the effect of PCL/COL scaffold and BBR/PCL/COL (25, 50, 75 and 100 μg/mL) scaffolds on osteogenic differentiation of DPSCs, we seeded DPSCs on the scaffolds and cultured with osteogenic induction. Figure 4A(a, b) was the results of alkaline phosphatase activity (ALP activity) on day 7 and 14. On day 7, the activity of ALP in BBR/PCL/COL scaffold groups were significantly higher than that in the PCL/COL scaffold group. However, the promoting effect was not concentration dependent. The content of ALP in all BBR/PCL/COL groups were higher than that in the PCL/COL scaffold on day 14, and the activity of ALP was most significant in the 50 μg/mL scaffold group (11.07 ± 0.32 U/mL) (*P* < 0.05). The results of ALP staining (Fig. S1 in the supplementary information) showed that compared with the PCL/COL scaffold, all BBR/PCL/COL scaffolds showed larger and darker purple-blue precipitates, of which 50 ug/mL BBR/ PCL/COL scaffold had the most prominent staining. Moreover, compared with the staining results of 7 days, significantly denser nodules were observed at 14 days in all groups. ALP staining results were consistent with ALP activity.

Moreover, we further used Real-time polymerase chain reaction (RT-PCR) to evaluate the expression level of osteogenic genes (ALP, BMP2, OCN and COL-1) of DPSCs, Fig. 4B. On day 7, 50 μg/mL scaffold group showed 1.78 ± 0.20-fold, 6.19 ± 0.23-fold higher expression of the genes associated with early differentiation of bone differentiation, ALP, COL-1, than the PCL/COL group. The expression of ALP in 75 μg/mL scaffold group also showed an up-fold of 1.57 ± 0.11-fold than control. The expression of BMP2 of DPSCs cultured on 50, 75 μg/mL scaffold group was respectively 7.03 ± 0.34-fold and 6.32 ± 0.55-fold higher than in the control. However, there was no statistical difference between the two groups. On day 14, the expression of ALP in all BBR/PCL/COL scaffolds were significantly increased (1.89 ± 0.15-fold, 3.63 ± 0.10-fold, 3.13 ± 0.34-fold and 1.97 ± 0.42-fold higher), and the 50, 75 μg/mL scaffold groups were more significantly than 25, 100 μg/mL groups (*P* < 0.05). BMP2 and Runx2 was increased in the 25, 50 and 75 μg /mL groups, with the most significant up-regulation in 50 μg/mL.

### Bone repair potential in vivo

**Figure 5:**
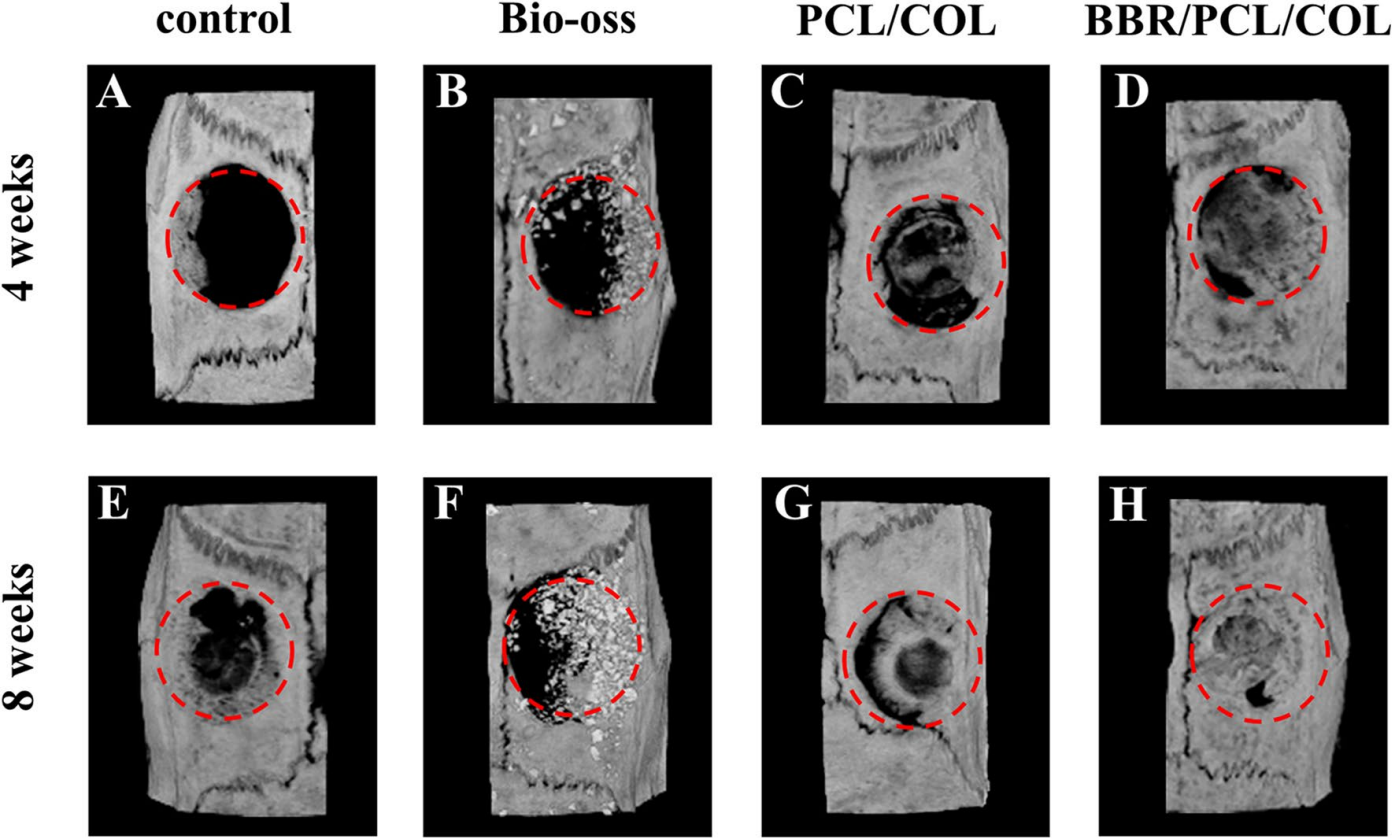
Images of micro-CT 3D reconstruction of rat calvaria defects at 4 weeks and 8 weeks after implantation.

**Figure 6:**
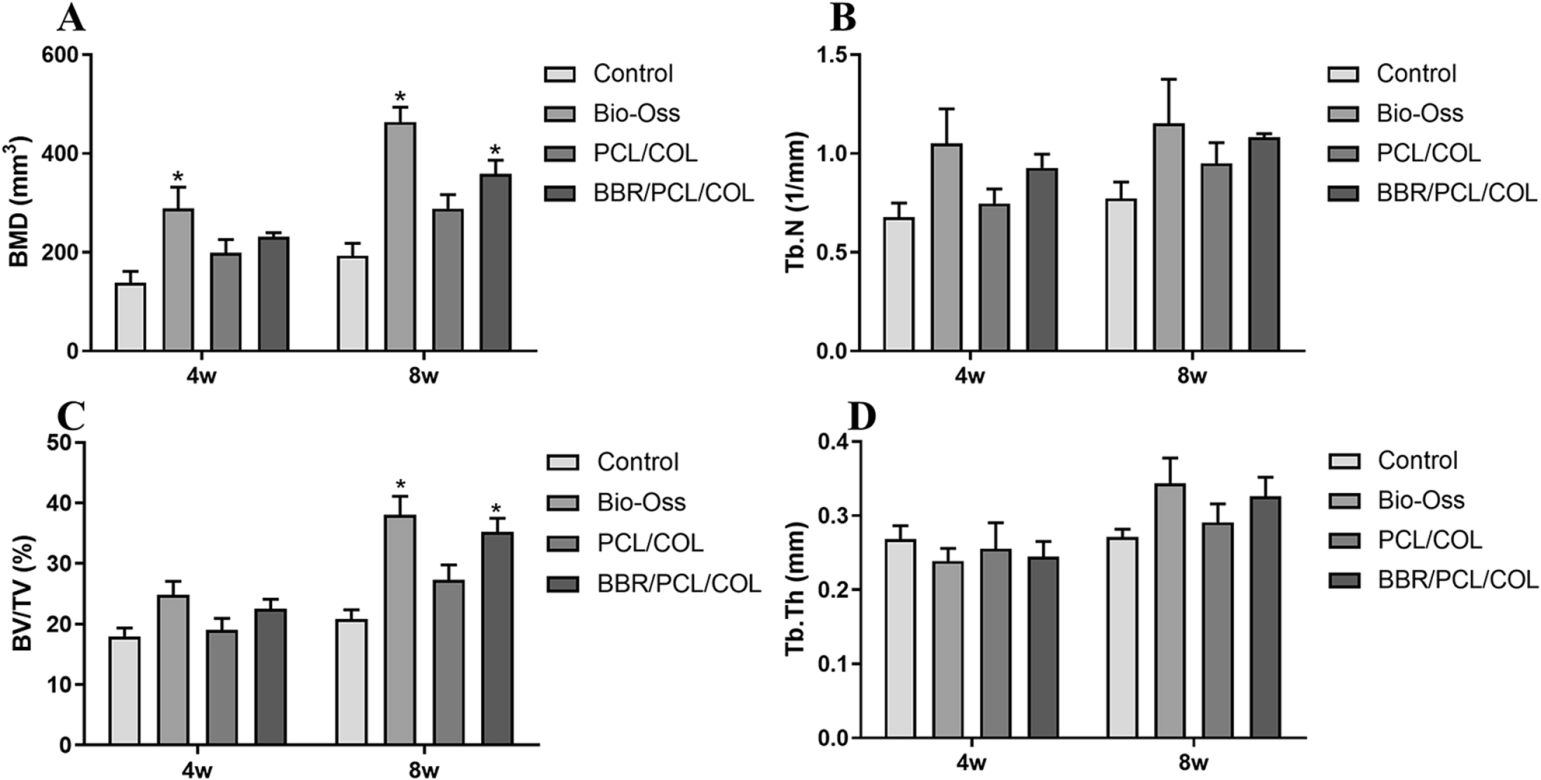
Quantitative analysis of bone related parameters at 4 weeks and 8 weeks after implantation. “*” means the comparison between the control group and the experimental group, *P* < 0.05.

To evaluate the in vivo bone repair ability of the scaffold, PCL/COL scaffold and BBR/PCL/COL scaffolds were implanted to the prepared critical bone defects. None of the rats showed significant inflammation or immune response after surgery. The skulls were harvested at 4 weeks and 8 weeks for micro-CT evaluation. Bone defect area was marked with dashed lines in Fig. 5. At 4 weeks, there was almost no new bone formation in the control group, and only a limited amount of mineralized deposits was found in the marginal area of the defect (Fig. 5A). In the Bio-oss group, the defect was filled with bone powder, part of the powder was displaced (Fig. 5B). The scaffolds provided support for new bone formation. In PCL/COL scaffold group, moderate mineral deposits were found in the center and edges of the defect (Fig. 5C). The mineralized area of BBR/PCL/COL scaffold group was larger than PCL/COL scaffold group (Fig. 5D). With time going on, the defect area was gradually repaired by newly formed minerals. At 8 weeks, the mineralization of the control group extended to the center, however the center of defect remained empty (Fig. 5E). In the Bio-oss group, the defect was still filled with bone powder, while the mineralization connected to the defect margin were more abundant (Fig. 5F). Compared with 4 weeks, the mineralization in the PCL/COL scaffold group increased significantly in 8 weeks, with limited defects still open (Fig. 5G). Surprisingly, the defect in the BBR/PCL/COL scaffold group was almost repaired by newly formed minerals (Fig. 5H). Meanwhile, in order to quantify the mineralization of newly formed tissue in the defect area, bone mineral density (BMD), bone volume/tissue volume (BV/TV), trabecular number (Tb. N) and trabecular thickness (Tb. Th) were analyzed (Fig. 6). At 8 weeks, compared to the control group, BBR/PCL/COL scaffold group showed higher bone mineral density (*P* = 0.03) and the BV/TV was also significantly increased (*P* = 0.03). At 8 weeks, the number of trabeculae and the thickness of trabeculae in the BBR/PCL/COL scaffold group increased compared to the control and PCL/COL scaffold group, although there was no significant difference. However, the results of Bio-oss group were false high due to the undegraded bone powder, which could not be avoided during measurement. Combined with the results of histological analysis, the high mineralization in the defect area was mostly caused by bone powder, and limited new bone was formed.

**Figure 7:**
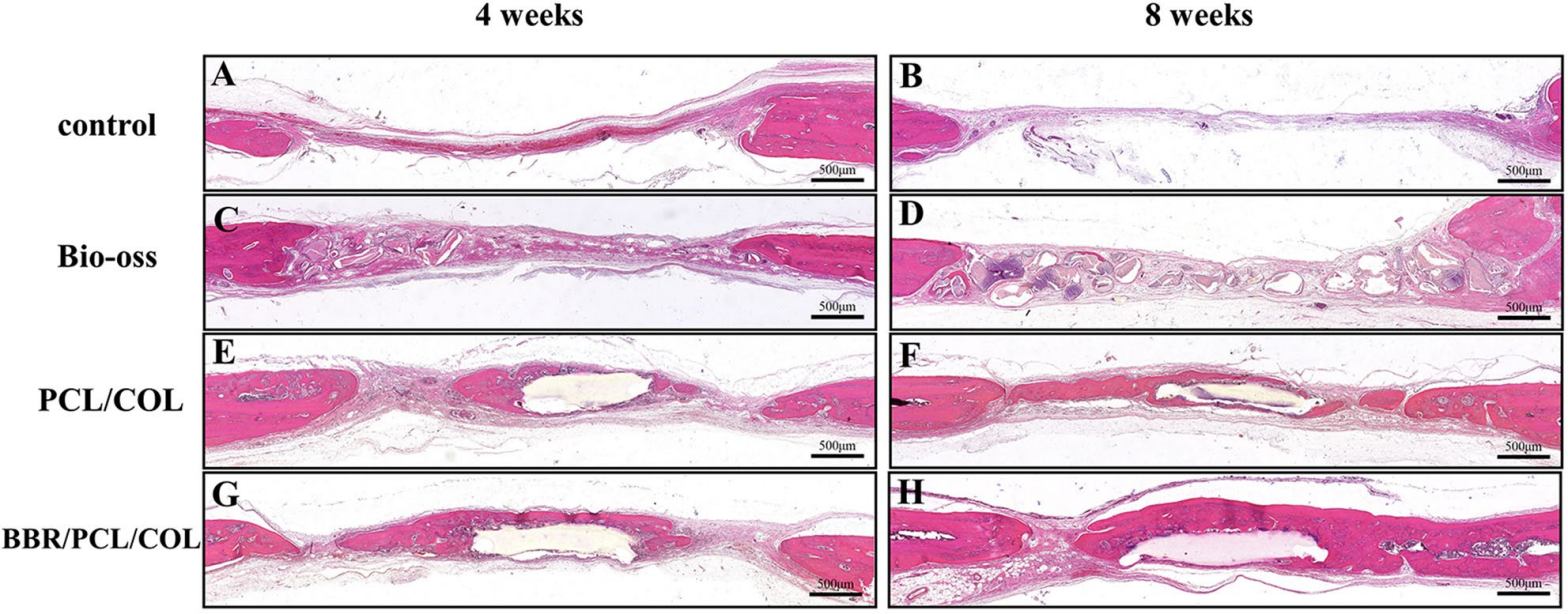
H&E staining of rat calvaria defects at 4 and 8 weeks after implantation.

**Figure 8:**
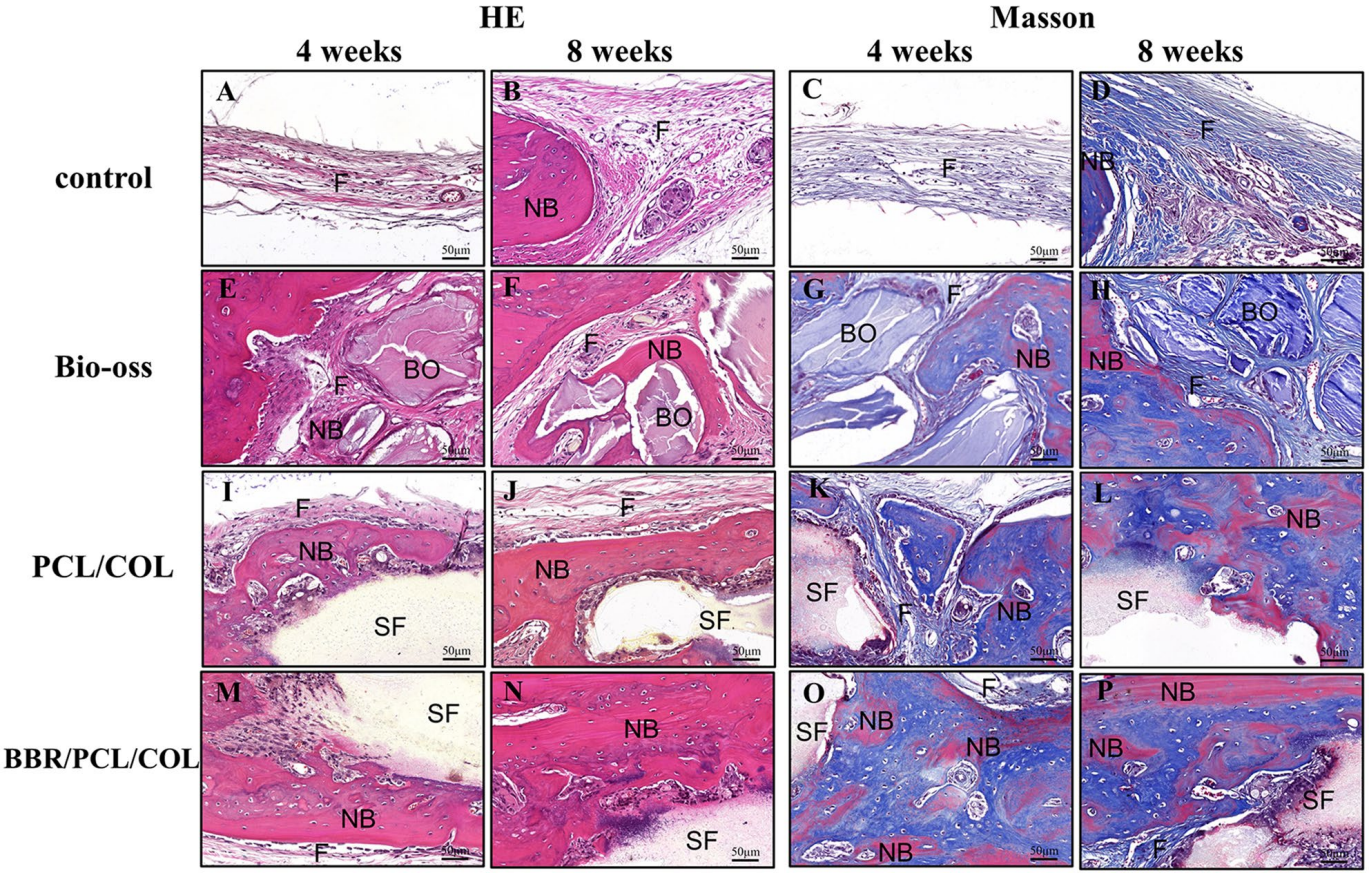
H&E and Masson staining of defects at higher magnification. F, fibrous tissue; BO, Bio-oss; NB, new bone; SF, scaffold.

H&E and Masson staining were used for histological analysis of bone formation. 4 weeks after implantation, the defect area in the control group was filled with thin connective fibrous tissue, and almost no newly formed bone was found (Fig. 7A). In the Bio-oss group, the defect area was filled with bone powder, which was wrapped by fibrous tissue, and bits of bone tissue was formed at the defect edge (Fig. 7C). In the PCL/COL and BBR/PCL/COL scaffold group, new bone was found to be deposited along the surface of the scaffold (Fig. 7E). Compared to the PCL/COL scaffold group, the BBR/PCL/COL scaffold group showed deeper H&E staining and thicker bone matrix (Fig. 7G). At 8 weeks, the defect in the control group was still dominated by fibrous tissue (Fig. 7B) and the bone powder in the Bio-oss group was still undegraded (Fig. 7D). PCL/COL and BBR/PCL/COL scaffold group showed more mature bone quality than 4 weeks (Fig. 7F, H). Further observation under the higher magnification, the control group was dominated by fibrous tissue, and a small amount of mineralized sediments appeared at 8 weeks (Fig. 8A, B). Bone powder in the Bio-oss group was surrounded by fibers (Fig. 8E, F). The PCL/COL and BBR/PCL/COL groups found abundant cellular infiltration and new bone was deposited along the scaffold (Fig. 8I, J). The BBR/PCL/COL group showed significant angiogenesis, some new bone and blood vessels extended into the scaffold, and osteocytes embedded in the bone matrix (Fig. 8M, N). Masson’s trichrome staining results distinguished fibrous tissue from new bone clearly. The control group showed only pale blue-stained fibrous tissue at both 4 weeks and 8 weeks (Fig. 8C, D); In the Bio-oss group, it was found that a large number of new fibrous tissues were surrounded by undegraded bone powder particles, and red mineralized new bone appeared at 8 weeks (Fig. 8G, H).); PCL/COL group (Fig. 8K, L) and BBR/PCL/COL group (Fig. 8O, P) showed abundant blue-stained fibrous connective tissue and some red-stained new bone, a large area of new bone was found in the BBR/PCL/COL group at 8 weeks (Fig. 8P)**.**

We further performed histomorphological analysis of the sections, BBR/PCL/COL scaffold group exhibited a significantly higher percentage of newly formed bone diameter (NBD) than the control group (*P* < 0.001), Bio-Oss group (*P* = 0.001) and PCL/COL scaffold group (*P* < 0.001) in 4 weeks. Similar results were found at 8 weeks (Table 1). The newly formed bone area (NBA) was calculated in the defect edge. The NBA of BBR/PCL/COL scaffold group was higher than the other groups, however the difference was only statistically significant when compared with the control (*P* < 0.001) (Table 2).

**Table 1:** Histomorphometric analysis results of percentage of newly formed bone diameter (NBD) (n = 6).

Time	Control^1^		Bio-Oss^2^	PCL/COL^3^	BBR/PCL/COL^4^	4 versus 1	4 versus 2	4 versus 3
	Mean (SD)		Mean (SD)	Mean (SD)	Mean (SD)	*P* value	*P* value	*P* value
4w	5.21 (1.88)	37.18 (8.68)		12.36 (7.58)	76.32 (6.04)	0.000*	0.001*	0.000*
8w	11.87 (4.41)	55.83 (11.45)		20.38 (3.21)	89.37 (3.32)	0.000*	0.001*	0.000*

*SD*: standard deviation.

1: Control; 2: Bio-Oss; 3: PCL/COL; 4: BBR/PCL/COL.

One-way analysis of variance.

*Statistically significant at *P* < 0.05.

**Table 2:** Histomorphometric analysis results of newly formed bone area in the defect edge (NBA, μm^2^) (n = 6).

Time	Control^1^	Bio-Oss^2^	PCL/COL ^3^	BBR/PCL/COL^4^	4 versus 1	4 versus 2	4 versus 3
	Mean (SD)	Mean (SD)	Mean (SD)	Mean (SD)	*P* value	*P* value	*P* value
4w	1,526.93 (505.99)	31,154.73 (8,033.86)	45,597.53 (8,225.50)	54,852.73 (5,899.71)	0.000*	0.38	1.000
8w	4,385.33 (1,590.78)	69,035.93 (11,683.85)	74,884.87 (6,373.69)	115,962.07 (21,867.70)	0.000*	0.38	0.75

*SD*: standard deviation.

1: Control; 2: Bio-Oss; 3: PCL/COL; 4: BBR/PCL/COL.

One-way analysis of variance.

*Statistically significant at *P* < 0.05.

## Discussion

The repair of large skeletal defects in maxillofacial region remains a serious challenge. The emerging tissue engineering technology provides a novel strategy for bone repair. In our study, we created a group of BBR/PCL/COL nanofibrous scaffold with BBR concentration of 25, 50, 75, 100 μg/mL respectively, which provided a microenvironment to promote osteogenic differentiation and accelerate bone defects repair.

PCL/COL and BBR/PCL/COL scaffolds with various BBR concentration were successfully fabricated by electrospinning. All groups of scaffolds exhibited a disordered fibrous structure, the average diameter of fibers decreased with the increase of BBR content. The morphology of BBR/PCL/COL scaffolds were similar to PCL/COL scaffold (Fig. 1A). We believed that the addition of BBR does not affect the electrospinning process.There are various factors which can influence electrospun fiber diameter, mainly divided to electrospinning parameters, solution and environmental parameters^25^. BBR increased the conductivity of the polymer solution, as the conductivity of the solution increased, the charge on the surface of the droplet increased, resulting in a decrease of fiber diameter^26^. Furthermore, BBR has a large charge density, and the large charge density increases the self-repulsion and the stretching force when droplet passes through the electrostatic field, also leads to the decrease of fiber diameter^27^. Our results are consistent with previous researchers^28,29^. With the fiber diameter decreasing, scaffold with higher porosity can be obtained. High-porosity scaffolds are conducive to the secretion of ECM, mimic physiological microenvironment, finally accelerate the bone repair process^30^.

The local release of the scaffold-loaded drug is crucial to provide a microenvironment favour of bone tissue repair. Pure berberine powder was difficult to applied in tissue engineering, so we loaded it to PCL/COL scaffold by electrospinning method and detected the drug cumulative release profile. Although an initially limited burst was found on the first day, the BBR/PCL/COL scaffolds could sustain release the drug for up to 27 days. When the surface of the scaffold in contacted with the release reagent, the loaded drug dissolved and released rapidly, resulting in an initial burst, drugs release in this way leads to explosive effects^31^. The limited diffusion of BBR from partially crystallized PCL leaded to the followed slow release rate^32^. With the degradation of polymer nanofibers, the scaffold structure changed dynamically over time, achieving the sustained release of BBR. Bone tissue engineering has certain requirement on scaffold degradation rate. The ideal degradation rate should be synchronized with the tissue regeneration rate^33^. In vivo experiment showed that at the time of 8 weeks, the shape of PCL/COL scaffold and BBR/PCL/COL scaffold were basically complete, with only a little degradation. The average degradation time of PCL homopolymer was 2–4 years^34^, COL degrades rapidly, disappears completely in several weeks. The participation of COL made it possible for the scaffold releasing BBR continuously. Moreover, the degradation time of scaffolds can also be controlled by adjusting the proportion of PCL and COL in electrospinning solution^35^. This property can expand the application of scaffolds, we will explore further in future studies.

BBR is widely used as an agent for digestive system diseases treatment such as bacillary dysentery and gastroenteritis. With the depth study of BBR, researchers found that BBR had a bone-protective effect. For example, Li et al^36^. reported that the principle of BBR to treat osteoporosis is achieved by enhancing bone density and inhibiting osteoclast activity. Our previous study found that BBR could restore the downregulation of osteogeneic-related genes expression in MSCs caused by *P*.gingivalis^37^. Most of the current researches focus on the influence of pure berberine agent on cells activity. However, in order to apply its bone-promoting properties to tissue engineering, BBR should be loaded into scaffold. Cai et al^38^. encapsulated BMP2 and BBR into microspheres and assembled to gel for bone repair, nonetheless they just concentrated on the antibacterial effects of BBR. Chen et al^39^. prepared a sodium hyaluronate and sodium alginate (HA/SA) scaffold loaded with BBR and found that BBR could activate Wnt/ β-catenin pathway to promote the cartilage regeneration. Similar to the results of BBR directly stimulating MSCs, our data indicates the BBR/PCL/COL scaffold promoted DPSCs osteogenic differentiation in vitro by up-regulating the expression of osteogenic-related genes, especially at the concentrations of 50 and 75 μg/mL. We also evaluated the osteogenic evaluation of BBR/PCL/COL in vivo. The results show that at 8 weeks, in the BBR/PCL/COL group, the defect area was basically repaired by newly formed bone-like tissue. In addition, BMD was significantly higher than other groups. Histological examination shows that the BBR/PCL/COL scaffold group showed denser H&E staining and thicker bone matrix, and the newly formed collagen was also evident. The results of our research strongly illustrate that BBR/PCL/COL scaffold could accelerate bone defect repair.

## Conclusion

BBR/PCL/COL electrospun scaffolds containing different concentrations of BBR (25, 50, 75 and 100 μg/mL) were successfully prepared via electrospinning technology, and their potential function to promoted the osteogenic differentiation of DPSCs was evaluated. In vivo results showed that the BBR/PCL/COL electrospun scaffold accelerates bone defect repair process. This suggests that BBR/PCL/COL electrospun scaffold may offer a potential treatment option for large skeletal defects repair.

## Methods

### Preparation of scaffold

Polycaprolactone (Mw = 160 kDa, Dai Gang Biology, China), Type I collagen (Sigma-Aldrich, USA), and Berberine (Sigma-Aldrich, USA) were used to prepare scaffolds. We fabricated BBR/PCL/COL scaffolds with different concentrations of BBR (25, 50, 75 and 100 μg/mL) and PCL/COL scaffolds through electrospinning. First, dissolved BBR powder in hexafluoroisopropanol (HFIP, Aladdin Industrial Corporation, Shanghai, China) to prepared a 0.1 mg/mL stock solution. Then diluted the stock solution to concentrations of 250, 500, 750 and 1000 μg/mL, respectively. Dissolved 1.5 g PCL and 0.9 g COL in 9 mL HFIP by magnetic stirring for 2 h. Then 1 mL of BBR solution of different concentrations was added to the above solution and magnetically stirred for 10 h. The final concentrations of the electrospinning solution were 25, 50, 75 and 100 μg/mL, respectively. Load the polymer solution into a 10 mL syringe (with a 0.34 mm metal needle) during the electrospinning process. The electrospinning device was purchased from Qingdao Junada Technology Co., Ltd. We set the flow rate at 1 mL/h, and the voltage applied was 12 kV. The collection distance (from the needle to the collecting aluminum foil) was 15 mm. All the scaffolds were freeze-dried for 12 h, sterilized by UV irradiation on each side for 4 h and sealed with sterile polythene bags for subsequent cell culture.

### Characterization of scaffold

Scanning electron microscope (SEM, SU3500, HITACHI, Japan) was applied to identify the morphology of the BBR/PCL/COL and PCL/COL scaffolds. The accelerating voltage applied was 2.0 kV. Energy dispersive x-ray spectroscopy (Aztec, OXFORD instrument, UK) was used to determine the elemental composition of the scaffolds. To analyse the hydrophilic/hydrophobic nature of the nanofibrous scaffolds, the water contact angles were tested by the contact angle measure instrument (Face-kyowa, Dropmaster, Japan). We recorded an average of three tests. X-ray diffraction (XRD, Edax, Phoenix, AZ, USA) was used to evaluate the phase composition and crystallinity of the scaffolds.

### Release of BBR from BBR/PCL/COL scaffolds

The standard curve of BBR was established by recording the absorbance values of berberine gradient dilutions at 344 nm. 10 mg of BBR/PCL/COL (25, 50, 75 and 100 μg/mL) scaffolds were immersed in 2 mL PBS buffer, and placed in a shaker incubator (100 rpm, 37 °C). The buffer was harvested at predetermined time points (1, 3, 5, 7, 11, 15, 21 and 27 days) and the same amount of liquid was replenished. The absorbance was measured at 344 nm with the microplate reader. The contents of BBR in buffer were calculated and the cumulative release curve was drawn.

### Isolation and culturing of DPSCs

The experiments were approved by Nanjing Stomatological Hospital Ethics Committee and all the processes were in compliance with relevant guidelines. Teeth pulp tissues were acquired from impacted third molars, donors aged 18–25 years, informed consent was obtained from all donors. The methods of isolation of DPSCs were as follows. In brief, the pulp tissue was removed from the tooth under sterile conditions, minced into small pieces about 0.5 × 0.5 mm^2^ and digested with 3 mg/mL collagenase type I and 4 mg/mL dispase for about 60 min at 37 °C. Then filtered the digested mixtures with a 70-mm cell strainer to obtain single-cell suspensions and centrifuged at 1000 rpm for 3 min. Cells were resuspended with DMEM containing 20% FBS and were transferred to a 25 mm^2^ tissue culture flask, incubated at 5% CO_2_ with 37 °C. Changed the culture medium every 3 days and the cells from passage 3–5 were used for further experiments.

### The morphology of DPSCs cultured on scaffolds

PCL/COL scaffolds and different BBR/PCL/COL (25, 50, 75 and 100 μg/mL) scaffolds were cut into circular plates (diameter 14 mm) and transferred to the bottom of the 24-well plate. The scaffolds were incubated in DMEM overnight before cell seeding. DPSCs with a density of 2 × 10^4^ cells were seeded in each well. For the immunocytochemical staining observation, the samples were fixed with 3.7% formaldehyde for 10 min, and permeabilized with 0.1% Triton X-100 for 5 min. The nucleus was counterstained by DAPI (Invitrogen, 0.5 mg/mL) and the cytoskeleton was stained by ActinRed (KeyGEN, 5U/mL). After rinsed, the samples were observed under the confocal microscope (Nikon A1, Japon). We further observed the morphology of DPSCs cultured on the scaffold with SEM, after coculture for 7 days, the samples were fixed with 2.5% glutaraldehyde for 30 min, progressively dehydrated in ethanol with 3 min for each step (30%, 50%, 70%, 80%, 90%, 95% and 100%). The sample was sprayed with gold on the surface, morphology of DPSCs grown on the scaffolds were observed by SEM under the voltage of 3.0 kV.

### The proliferation of DPSCs cultured on scaffolds

BBR/PCL/COL (25, 50, 75 and 100 μg/mL) scaffolds and PCL/COL scaffolds were put into 96-well plates with a diameter of 6 mm. DPSCs with a density of 5 × 10^3^ cells were seeded in each well. The cell counting kit-8 assay (cck8, CK04, Dojindo, Japan) were used to detect proliferation of DPSCs at day 1, 3, 5 and 7. The scaffolds were first rinsed with PBS buffer solution twice, then 100μL DMEM with 10% WST®-8 was added and incubated at 37 °C for 2 h. The absorbance value of each well at 450 nm was determined by the microplate reader.

### Osteogenic differentiation of DPSCs cultured on scaffolds

ALP Assay Kits (Beyotime, P0321, China) was applied to estimate the activity of alkaline phosphatase. Protein sample was collected using Cell lysis buffer (Beyotime, P0013J). The protein sample was reacted with the chromogenic substrate for about 5 min then added the reaction termination solution, and the absorbance at 405 nm was obtained to evaluate the acivity of ALP. ALP staining was used to detect mineral deposits in DPSCs. The samples were fixed with 4% paraformaldehyde for 15 min, then used the BCIP/NBT Alkaline Phosphatase Color Development Kit (Beyotime, China) for staining, and photographed with digital camera.

DPSCs with a density of 10^6^ cells per well were seeded on scaffolds in 6- well plates. After culture in osteogenic induction media for 7 days and 14 days, RT-PCR was used to determined the expression level of osteogenic-related genes of DPSCs. TRIzol (Invitrogen, Karlsruhe, Germany) was first used to isolate total RNA, then total RNA was reverse transcribed to cDNA. SYBR®Green Supermix (Bio-Rad Laboratories, Hercules, USA) was used for RT-PCR in the StepOne™ real-time PCR system (Applied Biosystems, Foster City, USA). Osteogenic marker genes ALP, BMP2, Runx2 and COL-1 were tested. All the sample were normalized to GAPDH. The primer sequences were summarized in Table 3.

**Table 3:** The primer sequences of RT-PCR analysis.

Genes	Forward primers	Reverse primers
ALP	ACCACCACGAGAGTGAACCA	CGTTGTCTGAGTACCAGTCCC
BMP2	ACCCGCTGTCTTCTAGCGT	TTTCAGGCCGAACATGCTGAG
Runx2	TGGTTACTGTCATGGCGGGTA	TCTCAGATCGTTGAACCTTGCTA
COL-1	GAGGGCCAAGACGAAGACATC	CAGATCACGTCATCGCACAAC
GAPDH	TGGCCTCCAAGGAGTAAGAA	TGTGAGGGAGATGCTCAGTG

### Rat critical bone defects and scaffolds implantation surgery

The experiments were approved by Nanjing Stomatological Hospital Animal Care and Use Committee, the care and use of animals were in compliance with requirements of relevant guidelines. According to the results in vitro, the 50 μg/mL BBR/PCL/COL scaffolds performed best in osteogenic induction differentiation, so we chosed this concentration for in vivo research. 24 male SD rats weighing about 250 g were selected and divided into four groups randomly (control, Bio-oss, PCL/COL, BBR/PCL/COL, for each group, n = 6). Rats were acclimatized 7 days and in abrosia 12 h before surgery. The rats were anesthetized with 2% pentobarbital sodium. A full-thickness incision of about 15 mm was carried out along the sagittal suture, then the calvarium was exposed by blunt separation. 6 mm diameter critical bone defects were made with a trephine on two sides of the sagittal suture. During the operation, the surgical area was continuously rinsed with saline. Then the scaffolds (PCL/COL, BBR/PCL/COL) with a diameter of 6 mm were implanted to cover the bone defect. The control group was left empty as negative control. In the Bio-oss group, the defect was filled with 0.04 g Bio-oss powder as positive control. Periosteum and skin were sutured with 4-0 sutures. The animals were sacrificed after 4 weeks and 8 weeks, and the calvaria was fixed in 10% formalin for the following evaluation.

### Micro-CT analysis

The micro-CT system (Skyscan 1176; Bruker, Germany) was obtained to evaluate bone repair in the defect area. The system was applied under the fixed parameters: X-ray voltage at 65 kV, current at 385 μA and the samples were scanned at an interval of 18 μm. Then bone related parameters were calculated in CTAn software.

### Histological staining

The calvaria were decalcified with 10% EDTA solution for 3 months then dehydrated and embedded in paraffin, prepared at a thickness of 5 μm. The slices were dyed by H&E staining and Masson staining respectively and further analyzed. Histomorphological analysis was carried out in CaseViewer 2.3 software. The following data was measured: (1) the diameter of total defect (DTD); (2) the diameter of newly formed bone (DNB); Percentage of newly formed bone diameter (NBD) % = (DNB / DTD) %; (3) newly formed bone area in the defect edge (NBA), NBA were measured in μm^2^.

### Statistical analysis

One-way analysis of variance (ANOVA) with Duncan’s test for multiple comparisons was conducted to evaluate differences between groups. Data were presented as mean ± standard deviation (n = 3). *P* < 0.05 was considered statisticant for all analyses.

## Supplementary Information

Supplementary Information.
