# Supplementary material for: Berberine-releasing electrospun scaffold induces osteogenic differentiation of DPSCs and accelerates bone repair
Source: Sci Rep. 2021 Jan 13;11:1027. doi: 10.1038/s41598-020-79734-9 (PMC7806735; doi:10.1038/s41598-020-79734-9)
Supplement: Supplementary file 1 — Supplementary Information. [file 41598_2020_79734_MOESM1_ESM.pdf]

Supplementary Information

Berberine-releasing Electrospun Scaffold Induces Osteogenic Differentiation of DPSCs and Accelerates Bone Repair

Lan Ma[1,†], Yijun Yu[1,†], Hanxiao Liu[1], Weibin Sun[2], Zitong Lin[3,*], Chao Liu[4,*] and Leiying Miao[1,*]

1 Department of Cariology and Endodontics, Nanjing Stomatological Hospital, Medical School of Nanjing University, Nanjing 210093, China;

2 Department of Periodontology, Nanjing Stomatological Hospital, Medical School of Nanjing University, Nanjing 210093, China;

3 Department of Dentomaxillofacial Radiology, Nanjing Stomatological Hospital, Medical School of Nanjing University, Nanjing 210093, China;

4 Department of Orthodontics, Nanjing Stomatological Hospital, Medical School of Nanjing University, Nanjing 210093, China.

*corresponding.

* Zitong Lin e-mail: linzitong710@163.com.

* Chao Liu e-mail: dxliuchao@163.com.

* Leiying Miao e-mail: miaoleiying80@163.com.

†these authors contributed equally to this work

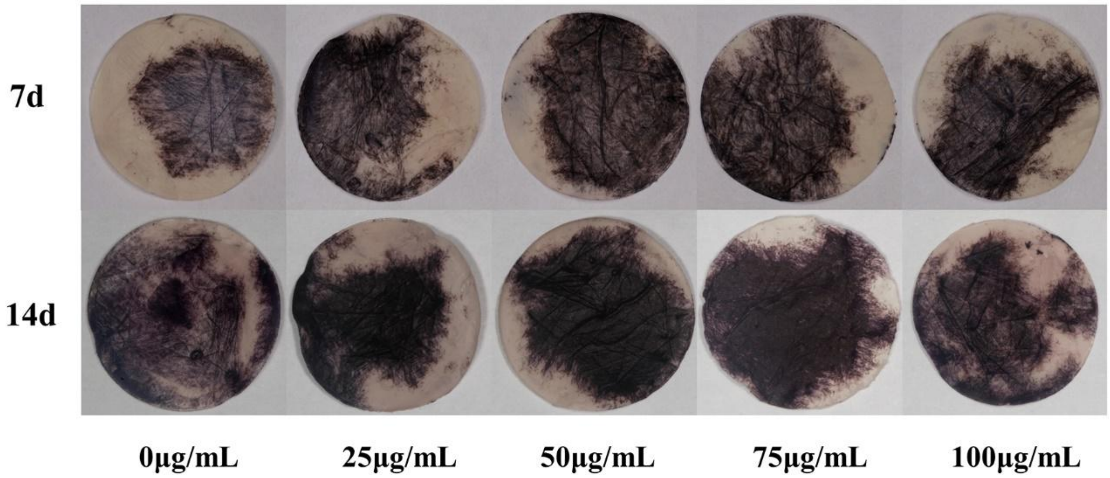

Supplementary Figure S1: ALP staining images of DPSCs co-cultured on scaffolds after 7 days and 14 days.

ALP staining results showed that compared with the PCL/COL scaffold, all BBR/PCL/COL scaffolds showed larger and darker purple-blue precipitates, of which 50 ug/mL BBR/ PCL/COL scaffold had the most prominent staining. Moreover, compared with the staining results of 7 days, significantly denser nodules were observed at 14 days in all groups. ALP staining results were consistent with ALP activity.
